# Profound neutralization evasion and augmented host cell entry are hallmarks of the fast-spreading SARS-CoV-2 lineage XBB.1.5

**DOI:** 10.1038/s41423-023-00988-0

**Published:** 2023-03-03

**Authors:** Markus Hoffmann, Prerna Arora, Inga Nehlmeier, Amy Kempf, Anne Cossmann, Sebastian R. Schulz, Gema Morillas Ramos, Luis A. Manthey, Hans-Martin Jäck, Georg M. N. Behrens, Stefan Pöhlmann

**Affiliations:** 1 Infection Biology Unit, German Primate Center, Kellnerweg 4, 37077 Göttingen, Germany; 2 Faculty of Biology and Psychology, Georg-August-University Göttingen, Wilhelmsplatz 1, 37073 Göttingen, Germany; 3 Department for Rheumatology and Immunology, Hannover Medical School, Carl-Neuberg-Straße 1, 30625 Hannover, Germany; 4 Division of Molecular Immunology, Department of Internal Medicine 3, Friedrich-Alexander University of Erlangen-Nürnberg, Glückstraße 6, 91054 Erlangen, Germany; 5 German Centre for Infection Research (DZIF), partner site Hannover-Braunschweig, Carl-Neuberg-Straße 1, 30625 Hannover, Germany; 6 Centre for Individualized Infection Medicine (CiiM), Feodor-Lynen-Straße 7, 30625 Hannover, Germany

**Keywords:** Immune evasion, Cell biology

Since late 2022, the share of infections caused by the SARS-CoV-2 lineage XBB.1.5 has gradually increased in the United States, resulting in XBB.1.5 becoming the dominating SARS-CoV-2 lineage in the United States and a similar trend is likely to soon take place also in European countries. However, information on the virological properties of XBB.1.5 is scarce. Here, we conducted an initial virological assessment of the SARS-CoV-2 XBB.1.5 lineage.

The SARS-CoV-2 XBB lineage possesses an extraordinarily high capacity for antibody evasion due to its unique set of S protein mutations [1–4]. However, this trait may have come at the cost of a moderately reduced host cell entry efficiency as suggested by recent in vitro data [1, 5], which may explain why infections caused by XBB sublineages only accounted for a small proportion of total SARS-CoV-2 infections in several countries (except India) so far (Fig. 1a). Recently, this trend has changed for the United States, where the share of infections caused by SARS-CoV-2 sublineage XBB.1.5 has gradually increased since late 2022, and XBB.1.5 now represents the dominating SARS-CoV-2 lineage (Fig. 1a). Moreover, although being presently detected at low frequencies only, a similar increase in the share of XBB.1.5-related infections is also observed for European countries (Fig. 1a). The XBB.1.5 S protein differs by only one mutation (S486P) from the S protein of the parental XBB.1 lineage, and this mutation is located in the receptor-binding domain (RBD) (Fig. 1b). Thus, it may affect transmissibility by modulating cell entry efficiency, and may alter sensitivity to antibody-mediated neutralization.

**Fig. 1:**
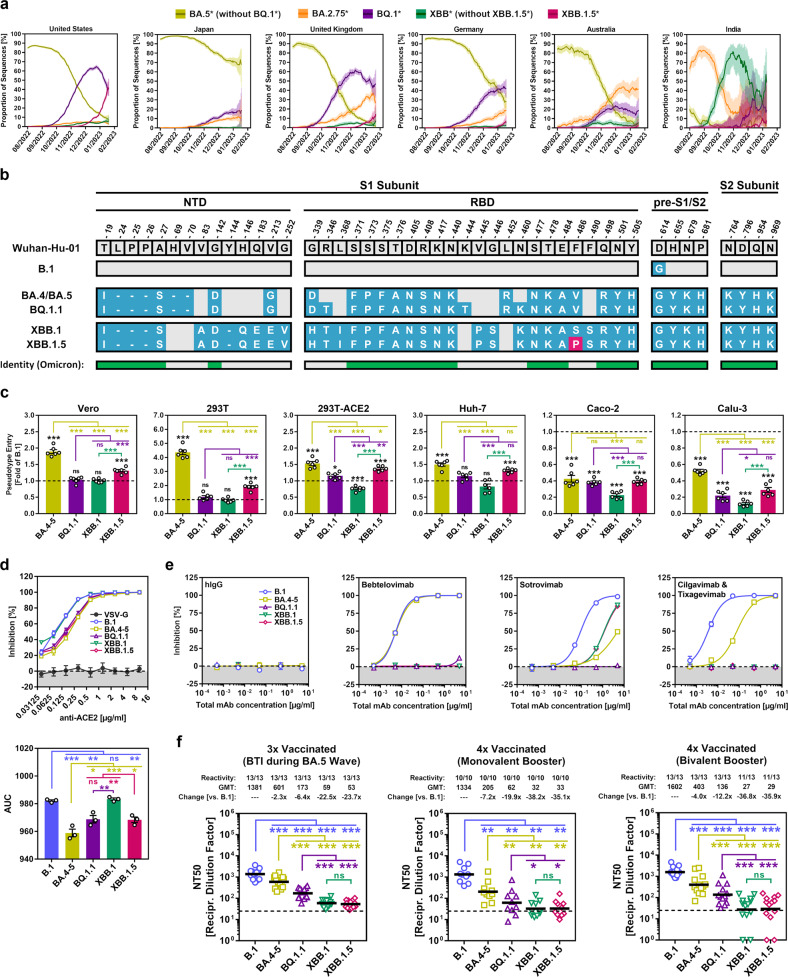
Host cell entry and neutralization sensitivity of the SARS-CoV-2 XBB.1.5 lineage. **a** Relative frequency of SARS-CoV-2 lineages BA.5* (without BQ.1*), BA.2.75*, BQ.1*, XBB* (without XBB.1.5*), and XBB.1.5* in selected countries (graphs are based on data retrieved from https://cov-spectrum.org/). **b** Mutations in the S proteins of SARS-CoV-2 lineages B.1, BA.4-5, BQ.1.1, XBB.1 and XBB.1.5 compared to the S protein of the Wuhan-Hu-01 isolate. The mutation highlighted in pink indicates the unique S protein mutation of the XBB.1.5 lineage that is not present in the S protein of the parental XBB.1 lineage. S protein mutations that are identical for all Omicron sublineages under study are indicated. NTD N-terminal domain, RBD receptor-binding domain, pre-S1/S2 region between RBD and the border between S1 and S2 subunits. **c** Cell line tropism and entry efficiency of the SARS-CoV-2 XBB.1.5 lineage. Identical volumes of pseudotype particles (pp) harboring the indicated SARS-CoV-2 S proteins were inoculated onto the indicated cell lines and pseudovirus entry was analyzed at 16–18 h postinoculation by measuring the activity of virus-encoded luciferase in cell lysates. Data represent the mean of six biological replicates (each performed with four technical replicates) and entry was normalized against B.1_pp_ (=1; indicated by dashed line). Error bars indicate the standard error of the mean (SEM). Statistical significance was analyzed by two-tailed Student’s *t*-tests with Welch correction (not significant [ns], *p* > 0.05; *p ≤ 0.05; **p ≤ 0.01; ***p ≤ 0.001). Please also see Supplementary Fig. S1. **d** Impact of antibody-mediated ACE2 blockade on host cell entry of the SARS-CoV-2 XBB.1.5 lineage. Pseudotype particles harboring the indicated SARS-CoV-2 S proteins or VSV-G (control) were inoculated onto Vero cells that had been preincubated with ACE2-blocking anti-ACE2 antibody. At 16–18 h postinoculation, pseudovirus entry was analyzed and normalized against samples without antibody (=0% inhibition). Data represent the mean of three biological replicates (performed with four technical replicates). Error bars indicate the SEM. The top graph shows dose-dependent inhibition of pseudovirus entry, while the bottom graph shows area under the curve (AUC) data. Statistical significance was analyzed by two-tailed Student’s *t*-tests with Welch correction (not significant [ns], *p* > 0.05; *p ≤ 0.05; **p ≤ 0.01; ***p ≤ 0.001). **e** Sensitivity of the SARS-CoV-2 XBB.1.5 lineage to neutralization by monoclonal antibodies (mAb). Pseudotype particles harboring the indicated SARS-CoV-2 S proteins were preincubated with individual mAb or mAb cocktails, and subsequently inoculated onto Vero cells. At 16–18 h postinoculation, pseudovirus entry was analyzed and normalized against samples without mAb (=0% inhibition). Data represent the mean of three biological replicates (performed with four technical replicates). Error bars indicate the SEM. **f** Sensitivity of the SARS-CoV-2 XBB.1.5 lineage to neutralization by antibodies induced by vaccination or vaccination plus breakthrough infection (BTI). Pseudotype particles harboring the indicated SARS-CoV-2 S proteins were preincubated with plasma from (i) three-times vaccinated individuals with BTI during the BA.5 wave in Germany (*n* = 13), (ii) four-times vaccinated individuals that received the monovalent BNT162b2/Comirnaty vaccine booster (*n* = 10), or (iii) four-times vaccinated individuals that received the bivalent BNT162b2/Comirnaty Original/Omicron BA.4-5 vaccine booster (*n* = 13). Following incubation, the samples were inoculated onto Vero cells. At 16–18 h postinoculation, pseudovirus entry was analyzed, normalized against samples without plasma (=0% inhibition), and the neutralizing titer 50 (NT50), indicating the plasma dilution resulting in half-maximal inhibition, was calculated. Data represent geometric mean NT50 values (geometric mean titers, GMT) from a single biological replicate (conducted with four technical replicates). Information above the graphs indicates responder rates (=proportion of plasma samples with detectable neutralizing activity), GMT, and the fold change in GMT for BA.4-5_pp_, BQ.1.1_pp_, XBB.1_pp_, or XBB.1.5_pp_ against B.1_pp_. Statistical significance was assessed by the Wilcoxon matched-pairs signed rank test (ns, *p* > 0.05; *p ≤ 0.05; **p ≤ 0.01; ***p ≤ 0.001). Please also see Supplementary Table S1 and Supplementary Fig. S2

Here, we performed an assessment on the host cell entry efficiency of the SARS-CoV-2 XBB.1.5 lineage and its sensitivity to antibody-mediated neutralization, using S protein-bearing pseudovirus particles (pp), which are a suitable model system for the analysis of SARS-CoV-2 host cell entry and its neutralization [6]. Particles pseudotyped with the S protein of the ancestral B.1 lineage (B.1_pp_) or Omicron sublineages BA.4/BA.5 (identical on amino acid level, BA.4-5_pp_), BQ.1.1 (BQ.1.1_pp_), or XBB.1 (XBB.1_pp_) were used for comparison. First, we compared cell line tropism and host cell entry efficiency of the different pseudoviruses. As expected, BA.4-5_pp_ displayed augmented cell entry efficiency compared to B.1_pp_ for most cell lines tested with the exception of TMPRSS2-positive Caco-2 (human, intestine) and Calu-3 (human, lung) cells [7], while cell entry of XBB.1.5_pp_ was significantly reduced compared to BA.5_pp_ [1] (Fig. 1c). Entry of BQ.1.1_pp_ was comparable or moderately increased relative to XBB.1_pp_, but reduced relative to BA.4-5_pp_ (with the exception of Caco-2 cells) (Fig. 1c). Importantly, XBB.1.5_pp_ showed significantly higher cell entry efficiency compared to XBB.1_pp_ for all cell lines tested (Fig. 1c). In order to investigate whether the increase in cell entry of XBB.1.5_pp_ relative to XBB.1_pp_ is the result of improved ACE2 usage, we limited ACE2 availability for cell entry using an ACE2-blocking antibody. We found that XBB.1.5_pp_ entered cells under conditions of limited ACE2 availability more efficiently than XBB.1_pp_ (Fig. 1d), suggesting that mutation S486P optimizes S protein-ACE2-interactions.

Since mutation S486P may also impact sensitivity to antibody-mediated neutralization, we further investigated whether currently used monoclonal antibody (mAb) therapies effectively neutralize XBB.1.5_pp_. In agreement with expectations, B.1_pp_ were effectively neutralized by Bebtelovimab, Sotrovimab, and a cocktail of Cilgavimab and Tixagevimab (Evusheld), while BA.4-5_pp_ were moderately resistant against neutralization by Sotrovimab and Cilgavimab/Tixagevimab, and BQ.1.1_pp_ could not be effectively neutralized by any mAb treatment tested (Fig. 1e) [8]. XBB.1_pp_ and XBB.1.5_pp_ displayed identical mAb neutralization profiles and only Sotrovimab showed neutralizing activity, which was moderately reduced compared to B.1_pp_ (Fig. 1e).

Finally, we investigated the neutralization sensitivity of XBB.1.5_pp_ to antibodies induced by vaccination with or without breakthrough infection (BTI). For this, we utilized plasma from triple-vaccinated individuals that experienced a BTI during the BA.5 wave in Germany, and plasma from quadruple-vaccinated individuals that received a monovalent or bivalent mRNA-vaccine booster as fourth vaccination. All tested plasma showed high neutralizing activity against B.1_pp_, while neutralizing activity against BA.4-5_pp_ and BQ.1.1_pp_ was moderately (BA.4-5_pp_: 2.3–7.2-fold reduced compared to B.1_pp_) or strongly (BQ.1.1_pp_: 6.4–19.9-fold reduced compared to B.1_pp_) reduced (Fig. 1f), as expected [9]. In line with published results, neutralizing activity against XBB.1_pp_ was even further reduced compared to BA.4-5_pp_ and BQ.1.1_pp_ (XBB.1_pp_: 22.5–38.2-fold reduced compared to B.1_pp_) [1–4], and neutralizing activity against XBB.1.5_pp_ was comparable to that of XBB.1_pp_ (XBB.1_pp_: 23.7–35.9-fold reduced compared to B.1_pp_) (Fig. 1f).

In summary, our results indicate that the apparently increased transmissibility of the SARS-CoV-2 XBB.1.5 lineage is (at least in part) the combined result of its profound neutralization resistance and the improved ACE2 usage due to S protein mutation S486P. In fact, a deep mutational scanning study using S proteins of SARS-CoV-2 lineages B.1, BA.1 and BA.2 suggests that mutation S486P enhances ACE2 binding affinity [10]. While Sotrovimab retains neutralizing activity against XBB.1 and XBB.1.5 and thus constitutes a treatment option for patients, novel mAbs need to be developed in order to be prepared for emerging XBB.1.5 sublineages that may harbor mutations that confer resistance against Sotrovimab.

## Supplementary information

Supplementary Material
