# Supplementary material for: Profound neutralization evasion and augmented host cell entry are hallmarks of the fast-spreading SARS-CoV-2 lineage XBB.1.5
Source: Cell Mol Immunol. 2023 Mar 3;20(4):419–22. doi: 10.1038/s41423-023-00988-0 (PMC9982771; doi:10.1038/s41423-023-00988-0)
Supplement: Supplementary file 1 — Supplementary Material [file 41423_2023_988_MOESM1_ESM.docx]

**Profound neutralization evasion and augmented host cell entry are hallmarks of the fast-spreading SARS-CoV-2 lineage XBB.1.5**

**Markus Hoffmann^1,2^, Prerna Arora^1,2^,** **Inga Nehlmeier^1^, Amy Kempf^1,2^, Anne Cossmann^3^, Sebastian R. Schulz^4^, Gema Morillas Ramos^3,5^, Luis A. Manthey^3^, Hans-Martin Jäck^4^, Georg M. N. Behrens^3,5,6^, Stefan Pöhlmann^1,2^**

^1^Infection Biology Unit, German Primate Center, Kellnerweg 4, 37077 Göttingen, Germany

^2^Faculty of Biology and Psychology, Georg-August-University Göttingen, Wilhelmsplatz 1, 37073 Göttingen, Germany

^3^Department for Rheumatology and Immunology, Hannover Medical School, Carl-Neuberg-Straße 1, 30625 Hannover, Germany.

^4^Division of Molecular Immunology, Department of Internal Medicine 3, Friedrich-Alexander University of Erlangen-Nürnberg, Glückstraße 6, 91054 Erlangen, Germany

^5^German Centre for Infection Research (DZIF), partner site Hannover-Braunschweig, Carl-Neuberg-Straße 1, 30625 Hannover, Germany.

^6^Centre for Individualized Infection Medicine (CiiM), Feodor-Lynen-Straße 7, 30625 Hannover, Germany.

Correspondence: Markus Hoffmann ([mhoffmann@dpz.eu](mailto:mhoffmann@dpz.eu))

# Methods

**Cell culture**

All cell lines were incubated at 37 °C in a humidified atmosphere containing 5% CO_2_. Vero (African green monkey kidney, female, kidney; CRL-1586, ATCC; RRID: CVCL 0574, kindly provided by Andrea Maisner), 293T (human, female, kidney; ACC-635, DSMZ; RRID: CVCL 0063), 293T-ACE2 (293T cells stably overexpressing human ACE2) (1) and Huh-7 cells (human, male, liver; JCRB Cat# JCRB0403; RRID: CVCL_0336, kindly provided by Thomas Pietschmann) were maintained in Dulbecco's modified Eagle medium (PAN-Biotech), supplemented with 10% fetal bovine serum (Biochrom), 100 U/ml of penicillin, and 0.1 mg/ml of streptomycin (PAN-Biotech). In addition, Calu-3 (human, male, lung; HTB-55, ATCC; RRID: CVCL_0609, kindly provided by Stephan Ludwig) and Caco-2 cells (human, male, colon; HTB-37, ATCC, RRID: CVCL_0025) were maintained in minimum essential medium (Thermo Fisher Scientific), supplemented with 10% fetal bovine serum (Biochrom), 100 U/ml penicillin and 0.1 mg/ml streptomycin (PAN-Biotech), 1x non-essential amino acid solution (from 100x stock, PAN-Biotech) and 1 mM sodium pyruvate (PAN-Biotech). Transfection of 293T cells was carried out by calcium phosphate precipitation. Cell lines were routinely tested for mycoplasma contamination. Further, all cell lines were validated by STR analysis, partial sequencing of the cytochrome c oxidase gene, microscopic examination, and/or according to their growth characteristics.

**Expression plasmids and sequence analysis**

S protein sequences and information on SARS-CoV-2 lineages was retrieved from GISAID (Global Initiative on Sharing All Influenza Data) (<https://gisaid.org/>) and CoV-Spectrum (<https://cov-spectrum.org/>) databases. Expression plasmids pCAGGS-DsRed (2), pCAGGS-VSV-G (3), pCG1-SARS-CoV-2 B.1 SΔ18 (codon-optimized, C-terminal truncation of 18 amino acid residues, GISAID Accession ID: EPI_ISL_425259) (1), pCG1-SARS-CoV-2 BA.4-5 SΔ18 (codon-optimized, C-terminal truncation of 18 amino acid residues, GISAID Accession ID: EPI_ISL_11550739 and EPI_ISL_12029894) (4) and pCG1-SARS-CoV-2 BQ.1.1 SΔ18 (codon-optimized, C-terminal truncation of 18 amino acid residues, GISAID Accession ID: EPI_ISL_14752457) (5) and pCG1-SARS-CoV-2 XBB.1 SΔ18 (codon-optimized, C-terminal truncation of 18 amino acid residues, GISAID Accession ID: EPI_ISL_15384151) (6) have been described before. In addition, the expression plasmid for SARS-CoV-2 XBB.1.5 SΔ18 (GISAID Accession ID: EPI_ISL_16239158), was generated by introduction of mutation S486P into plasmid pCG1-SARS-CoV-2 XBB.1 SΔ18 by overlap-extension PCR. Correctness of all S protein sequences was confirmed by Sanger sequencing using a commercial service (Microsynth SeqLab).

**Pseudovirus particle production and transduction of target cells**

Production of vesicular stomatitis virus pseudovirus particles harboring SARS-CoV-2 S proteins was performed according to a published protocol (7). In brief, 293T cells transfected to express the respective S protein, VSV-G or DsRed (negative control) were inoculated with VSV-G-transcomplemented VSV*ΔG(FLuc) (kindly provided by Gert Zimmer) (8) at a multiplicity of infection of 3. After 1h of incubation, the inoculum was removed and cells were washed with PBS. Thereafter, medium containing anti-VSV-G antibody (culture supernatant from I1-hybridoma cells; ATCC no. CRL-2700) was added to all cells except those expressing VSV-G (which received medium without antibody). Culture supernatants were harvested at 16-18 h post inoculation, clarified by centrifugation (4,000 x g, 10 min), and stored at ‑80 °C until further use. Target cells were seeded into 96-well plates and allowed to reach 50-90% confluency (except for neutralization assays, were confluent monolayers were inoculated with pseudovirus particles). For experiments addressing the inhibitory effect of antibody-mediated ACE2-blockade, target cells (Vero) were preincubated for 30 min at 37 °C with different dilutions of anti-ACE2 antibody (10108-MM36, SinoBiologicals) before equal volumes of pseudovirus particles were added. Cells were incubated for 16-18 h before transduction efficiency was determined by measuring the activity of virus-encoded luciferase in cell lysates. For this, the culture medium was removed and PBS containing 0.5% Triton X-100 (Carl Roth) was added to lyse the cells (30 min, room temperature). Lysates were transferred into white 96-well plates, mixed with firefly luciferase substrate (Beetle-Juice, PJK), and luminescence was recorded using a Hidex Sense plate luminometer (Hidex).

**Ethics committee approval and enrolment of study participants**

Plasma sample collection was conducted following approval by the research ethics committee of the Institutional Review Board of MHH (8973 BO K 2020). All participants provided written informed consent prior to the use of plasma samples for research. All participants are part of the COVID-19 Contact (CoCo) Study (German Clinical Trial Registry, DRKS00021152), which is an ongoing (start: March, 2020), prospective observational study monitoring anti-SARS-CoV-2 IgG and immune responses in health care professionals (HCP) at Hannover Medical School and individuals with potential contact to SARS-CoV-2 (9, 10). CoCo Study participants provide regularly blood samples for anti-spike IgG and anti-nucleocapsid (NCP) IgG assessment. In addition, self-reported information about COVID-19 vaccinations and SARS-CoV-2 infection history including symptoms were collected via standardized questionnaires.

**Plasma samples**

Individual plasma information is provided in the supplementary table. Samples were pre-screened for SARS-CoV-2 S1-specific IgG using the anti-SARS-CoV-2-QuantiVac-ELISA (IgG) (EUROIMMUN). In total, plasma samples derived from three cohorts were analyzed and all samples were collected within two months following the last immunization event (vaccination or breakthrough infection [BTI]): *cohort 1*, 3x Vaccinated + BTI (BA.5 Wave): Three-times-vaccinated individuals who experienced a BTI during the BA.5 wave in Germany (n = 13; age range 24-62 (median = 43 years); male to female ratio 2:11); *cohort 2*, 4x Vaccinated (Monovalent Booster): Four-times vaccinated individuals that received a monovalent booster vaccination (BNT162b2/Comirnaty vaccine) as fourth vaccine dose (n = 10; age range 27-65 (median = 48.5 years); male to female ratio 4:6); *cohort 3*, 4x Vaccinated (Bivalent Booster): Four-times vaccinated individuals that received a bivalent booster vaccination (BNT162b2/Comirnaty Original/Omicron BA.4-5 vaccine) as fourth vaccine dose (n = 13; age range 40-65 (median = 54 years); male to female ratio 4:9). SARS-CoV-2 infection-free history of cohorts 2 and 3 was confirmed by absence of anti-SARS-CoV-2 NCP IgG using the anti-SARS-CoV-2 ELISA (NCP) (EUROIMMUN).

**Neutralization assay**

Neutralization assays were performed according to an established protocol (11). In brief, pseudovirus particles bearing the respective S proteins were pre-incubated for 30 min at 37 °C with different dilutions of heat-inactivated (56 °C, 30 min) blood plasma (dilution range: 1:25 or 1:6,400), before being inoculated onto Vero cells. Following an incubation period of 16-18 h, neutralization efficiency was determined based on the relative inhibition of pseudovirus entry (cells transduced with pseudovirus particles that were incubated in the absence of plasma served as reference; = 0% inhibition). Plasma dilutions leading to half-maximal inhibition (neutralizing titer 50, NT50) were calculated using a non-linear regression model. Only samples yielding an NT50 value of at least 6.25 were considered positive, while samples yielding an NT50 of less than 6.25 were considered negative and assigned an NT50 value of 1.

**Data analysis**

Data analysis was performed using Microsoft Excel (part of Microsoft Office Professional Plus, version 2016, Microsoft Corporation) and GraphPad Prism version 8.3.0 (GraphPad Software). Statistical significance was assessed by two-tailed Student’s t-test with Welch corrections (cell entry, ACE2-blockade) or by Wilcoxon matched-pairs signed rank test Dunn's multiple comparisons test (neutralization). Only p values of 0.05 or lower were considered statistically significant (ns [not significant], p > 0.05; *, p ≤ 0.05; **, p ≤ 0.01; ***, p ≤ 0.001).

**Limitations of the study**

This study has several limitations. First, pseudovirus particles bearing SARS-CoV-2 S protein were employed to study SARS-CoV-2 host cell entry and neutralization. While pseudovirus particles were previously shown to faithfully recapitulate SARS-CoV-2 host cell entry and its neutralization, our data await formal confirmation with clinical SARS-CoV-2 isolates, which were not available to us. Second, sample sizes of the respective cohorts are relatively small and preclude investigation of potential differences in SARS-CoV-2 XBB.1.5 neutralization as a result of biological factors like age or gender. As a consequence, additional studies with larger cohorts are needed to address this topic. Third, since donor-matched samples were not available, the impact of mono- and bivalent vaccine boosters on SARS-CoV-2 XBB.1.5 neutralization could only be assessed indirectly. Fourth, as all plasma samples were collected within eight weeks post vaccination/infection we cannot make any statement on SARS-CoV-2 XBB.1.5 neutralization after extended time periods post vaccination/BTI.

# Supplementary references

1. Hoffmann M, Arora P, Gross R, Seidel A, Hornich BF, Hahn AS, et al. SARS-CoV-2 variants B.1.351 and P.1 escape from neutralizing antibodies. Cell. 2021;184(9):2384-93 e12.

2. Hoffmann M, Kleine-Weber H, Schroeder S, Kruger N, Herrler T, Erichsen S, et al. SARS-CoV-2 Cell Entry Depends on ACE2 and TMPRSS2 and Is Blocked by a Clinically Proven Protease Inhibitor. Cell. 2020;181(2):271-80 e8.

3. Brinkmann C, Hoffmann M, Lubke A, Nehlmeier I, Kramer-Kuhl A, Winkler M, et al. The glycoprotein of vesicular stomatitis virus promotes release of virus-like particles from tetherin-positive cells. PLoS One. 2017;12(12):e0189073.

4. Arora P, Kempf A, Nehlmeier I, Schulz SR, Cossmann A, Stankov MV, et al. Augmented neutralisation resistance of emerging omicron subvariants BA.2.12.1, BA.4, and BA.5. Lancet Infect Dis. 2022;22(8):1117-8.

5. Arora P, Kempf A, Nehlmeier I, Schulz SR, Jack HM, Pohlmann S, et al. Omicron sublineage BQ.1.1 resistance to monoclonal antibodies. Lancet Infect Dis. 2023;23(1):22-3.

6. Arora P, Cossmann A, Schulz SR, Ramos GM, Stankov MV, Jack HM, et al. Neutralisation sensitivity of the SARS-CoV-2 XBB.1 lineage. Lancet Infect Dis. 2023;23(2):147-8.

7. Kleine-Weber H, Elzayat MT, Wang L, Graham BS, Muller MA, Drosten C, et al. Mutations in the Spike Protein of Middle East Respiratory Syndrome Coronavirus Transmitted in Korea Increase Resistance to Antibody-Mediated Neutralization. J Virol. 2019;93(2).

8. Berger Rentsch M, Zimmer G. A vesicular stomatitis virus replicon-based bioassay for the rapid and sensitive determination of multi-species type I interferon. PLoS One. 2011;6(10):e25858.

9. Barros-Martins J, Hammerschmidt SI, Cossmann A, Odak I, Stankov MV, Morillas Ramos G, et al. Immune responses against SARS-CoV-2 variants after heterologous and homologous ChAdOx1 nCoV-19/BNT162b2 vaccination. Nat Med. 2021;27(9):1525-9.

10. Behrens GMN, Cossmann A, Stankov MV, Schulte B, Streeck H, Forster R, et al. Strategic Anti-SARS-CoV-2 Serology Testing in a Low Prevalence Setting: The COVID-19 Contact (CoCo) Study in Healthcare Professionals. Infect Dis Ther. 2020;9(4):837-49.

11. Arora P, Zhang L, Rocha C, Sidarovich A, Kempf A, Schulz S, et al. Comparable neutralisation evasion of SARS-CoV-2 omicron subvariants BA.1, BA.2, and BA.3. The Lancet Infectious diseases. 2022;22(6):766-7.

# Supplementary Tables

**Table S1: Plasma information**

| **Cohort** | **ID** | **Gender** | **Age**  **(years)** | **V1/V2/V3** | **Post V3 BTI**  **(yes/no)** | **V4** | **Time between sampling and last vaccination (days)** | **Time between sampling and positive PCR test last vaccination (days)** | **Anti-SARS-CoV-2 S1 IgG**  **(BAU/ml)** |
| --- | --- | --- | --- | --- | --- | --- | --- | --- | --- |
| **1** | **9004** | **Male** | **36** | **BNT/BNT/MOD** | **yes**  **(BA.5 wave)** | **no** | **223** | **25** | **6567** |
| **1** | **9027** | **Female** | **24** | **AZ/BNT/BNT** | **yes**  **(BA.5 wave)** | **no** | **219** | **10** | **4662** |
| **1** | **9029** | **Female** | **29** | **BNT/BNT/BNT** | **yes**  **(BA.5 wave)** | **no** | **214** | **22** | **6618** |
| **1** | **9061** | **Female** | **55** | **AZ/AZ/BNT** | **yes**  **(BA.5 wave)** | **no** | **263** | **31** | **5452** |
| **1** | **9063** | **Female** | **62** | **AZ/BNT/BNT** | **yes**  **(BA.5 wave)** | **no** | **273** | **41** | **4891** |
| **1** | **9071** | **Female** | **46** | **AZ/AZ/MOD** | **yes**  **(BA.5 wave)** | **no** | **249** | **49** | **5499** |
| **1** | **9072** | **Female** | **31** | **AZ/BNT/BNT** | **yes**  **(BA.5 wave)** | **no** | **274** | **36** | **2392** |
| **1** | **9092** | **Male** | **43** | **AZ/BNT/BNT** | **yes**  **(BA.5 wave)** | **no** | **261** | **16** | **5495** |
| **1** | **9099** | **Female** | **39** | **AZ/AZ/BNT** | **yes**  **(BA.5 wave)** | **no** | **279** | **33** | **6023** |
| **1** | **9108** | **Female** | **53** | **BNT/BNT/MOD** | **yes**  **(BA.5 wave)** | **no** | **253** | **15** | **4515** |
| **1** | **9112** | **Female** | **53** | **BNT/BNT/BNT** | **yes**  **(BA.5 wave)** | **no** | **280** | **31** | **6801** |
| **1** | **9142** | **Female** | **34** | **AZ/BNT/BNT** | **yes**  **(BA.5 wave)** | **no** | **284** | **53** | **2922** |
| **1** | **9144** | **Female** | **50** | **AZ/AZ/MOD** | **yes**  **(BA.5 wave)** | **no** | **275** | **31** | **7392** |
| **2** | **8221** | **Male** | **48** | **AZ/AZ/BNT** | **no** | **yes**  **(BNT)** | **30** | **n.a.** | **1826** |
| **2** | **8383** | **Male** | **44** | **BNT/BNT/BNT** | **no** | **yes**  **(BNT)** | **37** | **n.a.** | **8018** |
| **2** | **8391** | **Female** | **38** | **BNT/BNT/BNT** | **no** | **yes**  **(BNT)** | **40** | **n.a.** | **2341** |
| **2** | **8808** | **Female** | **59** | **AZ/BNT/BNT** | **no** | **yes**  **(BNT)** | **7** | **n.a.** | **4709** |
| **2** | **8830** | **Male** | **65** | **AZ/AZ/BNT** | **no** | **yes**  **(BNT)** | **9** | **n.a.** | **1931** |
| **2** | **8864** | **Female** | **49** | **BNT/BNT/BNT** | **no** | **yes**  **(BNT)** | **46** | **n.a.** | **7836** |
| **2** | **9292** | **Female** | **31** | **BNT/BNT/BNT** | **no** | **yes**  **(BNT)** | **51** | **n.a.** | **5424** |
| **2** | **9310** | **Male** | **53** | **BNT/BNT/BNT** | **no** | **yes**  **(BNT)** | **2** | **n.a.** | **4990** |
| **2** | **9351** | **Female** | **27** | **BNT/BNT/BNT** | **no** | **yes**  **(BNT)** | **29** | **n.a.** | **11368** |
| **2** | **9357** | **Female** | **58** | **BNT/BNT/BNT** | **no** | **yes**  **(BNT)** | **33** | **n.a.** | **1668** |
| **3** | **9387** | **Female** | **59** | **BNT/BNT/BNT** | **no** | **yes**  **(BNT_bivalent_)** | **27** | **n.a.** | **6161** |
| **3** | **9445** | **Male** | **65** | **AZ/AZ/BNT** | **no** | **yes**  **(BNT_bivalent_)** | **27** | **n.a.** | **7177** |
| **3** | **9446** | **Female** | **61** | **BNT/BNT/BNT** | **no** | **yes**  **(BNT_bivalent_)** | **27** | **n.a.** | **5104** |
| **3** | **9448** | **Female** | **56** | **BNT/BNT/BNT** | **no** | **yes**  **(BNT_bivalent_)** | **11** | **n.a.** | **7945** |
| **3** | **9452** | **Male** | **44** | **no information** | **no** | **yes**  **(BNT_bivalent_)** | **27** | **n.a.** | **12697** |
| **3** | **9476** | **Female** | **51** | **no information** | **no** | **yes**  **(BNT_bivalent_)** | **33** | **n.a.** | **5429** |
| **3** | **9481** | **Female** | **58** | **no information** | **no** | **yes**  **(BNT_bivalent_)** | **23** | **n.a.** | **8758** |
| **3** | **9486** | **Female** | **52** | **no information** | **no** | **yes**  **(BNT_bivalent_)** | **23** | **n.a.** | **8519** |
| **3** | **9488** | **Male** | **54** | **no information** | **no** | **yes**  **(BNT_bivalent_)** | **26** | **n.a.** | **2644** |
| **3** | **9491** | **Female** | **46** | **no information** | **no** | **yes**  **(BNT_bivalent_)** | **33** | **n.a.** | **4773** |
| **3** | **9493** | **Female** | **61** | **no information** | **no** | **yes**  **(BNT_bivalent_)** | **28** | **n.a.** | **4819** |
| **3** | **9494** | **Female** | **50** | **no information** | **no** | **yes**  **(BNT_bivalent_)** | **33** | **n.a.** | **3610** |
| **3** | **9496** | **Male** | **40** | **BNT/BNT/MOD** | **no** | **yes**  **(BNT_bivalent_)** | **33** | **n.a.** | **8194** |

Cohorts: Cohort 1, 3x Vaccinated with BTI (BA.5 Wave); Cohort 2, 4x Vaccinated (Monovalent Booster); Cohort 3, 4x Vaccinated (Bivalent Booster).

SARS-CoV-2 infection-free status of cohorts “4x Vaccinated (Monovalent Booster)” and “4x Vaccinated (Bivalent Booster)” was confirmed by ELISA (anti-NCP-negative).

Abbreviations: ID, identifier; V#, vaccination #; AZ, AZD1222/Vaxzevria; BNT, BNT162b2/Comirnaty; MOD, mRNA-1273/Spikevax; BNT_bivalent_, Comirnaty Original/Omicron BA.4-5; BAU, binding antibody units; BTI, breakthrough infection; n.a., not applicable.

# Supplementary Figures

**Supplementary Figure 1: Cell line tropism and entry efficiency of the SARS-CoV-2 XBB.1.5 lineage**.


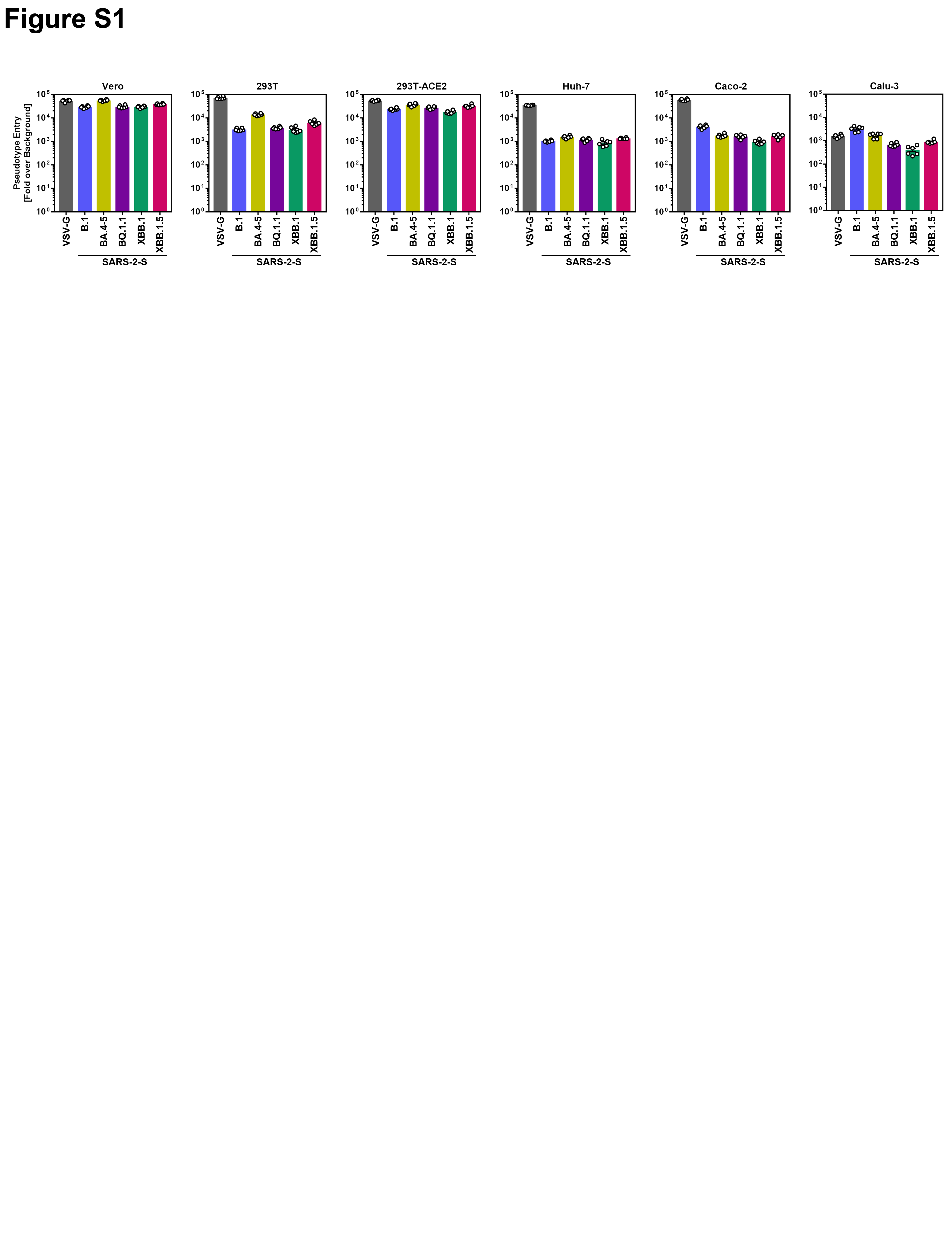


Identical volumes of pseudotype particles (pp) harboring the indicated SARS-CoV-2 S proteins or vesicular stomatitis glycoprotein (VSV-G) were inoculated onto the indicated cell lines and pseudovirus entry was analyzed at 16-18 h postinoculation by measuring the activity of virus-encoded luciferase in cell lysates. Data represent the mean of six biological replicates (performed with four technical replicates). Error bars indicate the standard error of the mean (SEM). Data normalized against the assay background (signals obtained for cells incubated with pseudovirus particles bearing no viral glycoprotein; given as fold over background).


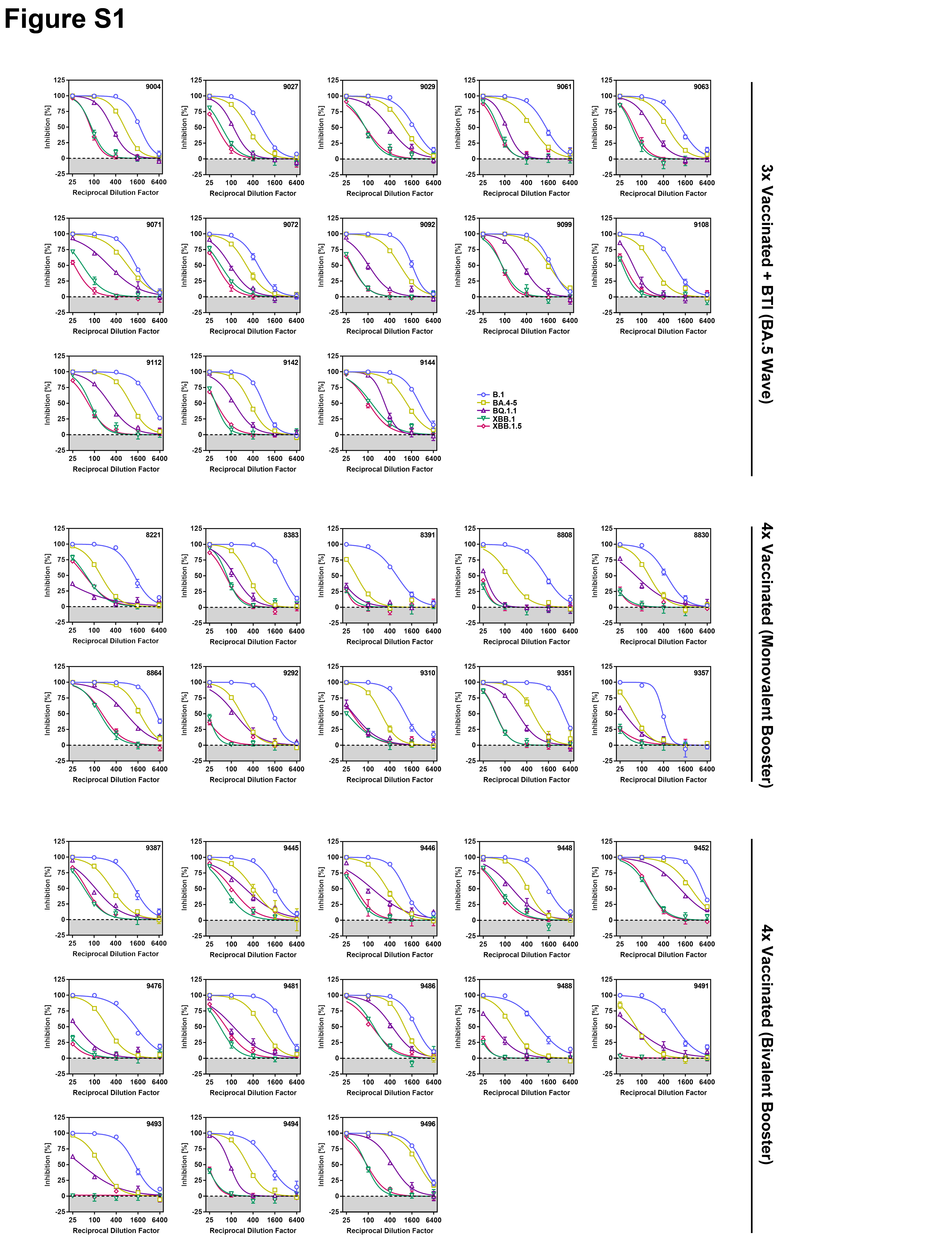


**Supplementary Figure 2: Sensitivity of the SARS-CoV-2 XBB.1.5 lineage to neutralization by antibodies induced by vaccination or vaccination plus breakthrough infection**.

Individual plasma neutralization data.
